# Impact of Race/Ethnicity on Pain Management Outcomes in a Community-Based Teaching Hospital Following Inpatient Palliative Care Consultation

**DOI:** 10.7759/cureus.823

**Published:** 2016-10-10

**Authors:** Duc Chung, Austin Sue, Susan Hughes, James Simmons, Tegest Hailu, Christine Swift, Patrick Macmillan

**Affiliations:** 1 Hospice and Palliative Medicine, University of California, San Francisco – Fresno Department of Family & Community Medicine; 2 University of California, San Francisco – Fresno Department of Family & Community Medicine

**Keywords:** pain, end-of-life care, race/ethnicity, health-care disparities, palliative care

## Abstract

**Objective:**

To examine race/ethnicity differences in pain management outcomes following inpatient palliative care consultation.

**Methods:**

We conducted a retrospective study based on data from a community-based teaching hospital in Fresno, CA, USA, from April 2014 to July 2015. One hundred sixty-one patients with life-limiting diagnoses and palliative care pain-related consultations were included. The patients were categorized into four racial groups: Caucasians, African-Americans, Hispanics, and Asians/Others. Demographics and baseline pain scores using the Visual Analogue Scale (VAS) were obtained. The outcome measures included the length of stay, time to consult, and pain scores at admission, 24 hours before the consultation, 24–48 hours after consultation, and at discharge.

**Results:**

The initial median pain scores were not significantly different between the groups, however, the Asians had slightly higher pain scores compared to the other groups. African-Americans, Caucasians, and Hispanics had significant differences in pain outcomes after consultation compared to 24 hours prior to consultation (p = 0.01, p < 0.01 and p = 0.02, respectively). Caucasians and Hispanics had significant differences in pain outcomes after palliative consultation compared to initial admission assessment (p < 0.01). The differences between discharge and admission pain scores were significant only for Asians, Caucasians, and Hispanics (p = 0.04, p < 0.01, p < 0.01, respectively) but not African-Americans. There were no significant pain score differences across the racial groups following consultations.

**Conclusion:**

There were no significant differences in pain reduction amongst the racial groups, suggesting that pain can adequately be managed in individual racial groups after inpatient palliative care consultations.

## Introduction

Pain has been described as the fifth vital sign. Since this advent, nurses and physicians have increasingly focused on treating pain in the inpatient setting. Sixty-two percent of patients with a cancer diagnosis experience pain. This percentage increases to almost all patients as they reach the terminal stages of their diagnoses [1-2]. Palliative care has become integral in the treatment of pain [3]. It is well documented that earlier intervention by the palliative care team improves symptom management, helps with appropriate discharge and decreases the length of stay in terminally ill patients [4-5]. Up to 90% of patients with cancer-related pain can be managed effectively, often with the assistance of a palliative care team. Researchers found that 75% of patients in their study had pain reduction when palliative care services were involved [6-7].

Likewise, patients with increased pain in the inpatient setting may have longer lengths of stay due to inadequate pain control. The patients referred to palliative care services within the first week of admission proved to have a shorter length of stay and lower in-hospital mortality compared to the patients who received consults after the first week of stay [8].

Despite diverse studies on the benefit of inpatient palliative care consultations, there have been mixed findings with regards to the effect of race on pain outcomes. A study by Ng, et al. demonstrated that Caucasians were more likely to require more morphine compared to Asians and Hispanics following limb fractures [9]. Furthermore, in studying pain outcomes amongst cancer, non-cancer, and surgical patients, Bell, et al. showed that Asians in general reported lower final pain severity compared to Caucasians when controlled for age, gender, Karnofsky score, the pre-consultation length of stay, and initial pain severity [10]. By contrast, Laguna, et al. found that seriously ill patients of Hispanic origin were more likely than Caucasians to report experiencing pain at hospital discharge and follow-up visits following inpatient palliative care consultations [6].

This study examines racial differences in pain management outcomes following inpatient palliative care consultations.

## Materials and methods

A retrospective study was conducted following Community Medical Centers institutional review board (IRB) approval. The study was based on chart review of data drawn from a community-based teaching hospital, Community Regional Medical Center (CRMC), in Fresno, CA from April 2014 to July 2015. The patients were identified from the records kept by the palliative care nurse manager. The following data were extracted: medical record number (MRN), date of admission, date/time of consult, date palliative consult completed, primary diagnoses, and date of discharge.

Further information was obtained from CRMC EPIC™ electronic medical records (Epic Systems, WI, USA). This information included: date of birth, gender, race/ethnicity, insurance, disposition, and pain scores. Palliative care consultations with pain measures were included using the Visual Analog Score (VAS; 0-10: 0 is no pain; 10 is a severe pain). Excluded were patients who did not have pain-related palliative care consultations or did not have reported pain scores at the established time points examined in the study.

The patients were categorized into four racial groups: Caucasians, African-Americans, Hispanics, and Asians/Others. Four pain scores were created by averaging all pain scores over the following specific 24-hour periods: first 24-hour period in the hospital (including admission), pain scores from the 24-hour period before the date/time of consult, pain scores 24-48 hours after consults were completed, and finally pain scores 24 hours prior to discharge. Age at time of admission, days from admission to time of initial consult, and total days in the hospital were also calculated.

## Results

### Demographics

Of the 1,671 palliative care consults, 197 pain consults were completed on 161 patients with life-limiting diagnoses of cancer or non-cancer (chronic obstructive pulmonary disease (COPD), congestive heart failure (CHF), liver failure and another end-organ failure). Of the 161 patients that met inclusion criteria, 15 were African-American, 15 were Asians/Others, 78 were Caucasian, and 53 were Hispanic. There was a higher percentage of males compared to females in the African-American, Caucasian, and Hispanic groups. The mean age was significantly different with Asians and Caucasians slightly older (p < 0.001). Furthermore, significant differences in the insurance status were observed, with a preponderance of the groups having Medicaid as a primary insurance (p = 0.003). The majority of patients had cancer compared to non-cancer diagnoses. The initial median pain score, although not statistically significant across the groups did show Asians had a slightly higher pain score (6.1) on the VAS compared to other groups. The majority of patients within all groups were discharged to home after hospitalization. A greater percentage of Asians and Caucasians was discharged either to home or skilled nursing facility with hospice. Higher percentages of deaths were seen in African-Americans and Caucasians compared to other groups. Significant differences in the length of stay were observed amongst the groups, with Hispanics having the longest length of stay, followed by Caucasians, Asians, and African-Americans (p = 0.02). There were no significant differences amongst the groups in days of admission to consult [See Table 1].

**Table 1:** Race/Ethnicity Group Characteristics

Characteristic	African-American	Asian/Other	Caucasian	Hispanic/Latino	p-value
Sample size (N)	15	15	78	53	
Gender (%): Male, Female	67,33	27,73	56,44	53,47	0.13
Age: Mean years (SD)	59 (14)	64 (15)	62 (14)	51 (15)	<0.001
Insurance (%): Medicaid, Medicare, Other	60,33,7	33,40,27	38,35,27	74,13,13	0.003
Diagnosis (%): Cancer, Non-cancer	87,13	87,13	83,17	89,11	0.86
Admission pain via VAS: Median (Q1 - Q3)	5.7 (3.9 – 7.9)	6.1 (1.6 – 6.5)	5.3 (3.9 – 7.2)	5.6 (3.7 – 7.2)	0.98
Disposition (%): Died, Home, Hospice, Other	27,33,27,13	7, 47,47,0	18,42,33,6	13,60,15,11	0.07
Length of stay: Days (Q1 – Q3)	7 (3 – 15)	8 (2 – 9)	5 (2 – 8)	7 (1 – 15)	0.02
Admission to Palliative Consult: Days (Q1 – Q3)	2 (1 – 13)	4 (1 – 6)	2 (1 – 6)	4 (1 – 7)	0.61

Note Table 1: Bolded values are significant at α = 0.05 level, SD is standard deviation, Q1 is the value in the 25th percentile, Q3 is the value in the 75th percentile.

**Figure 1:**
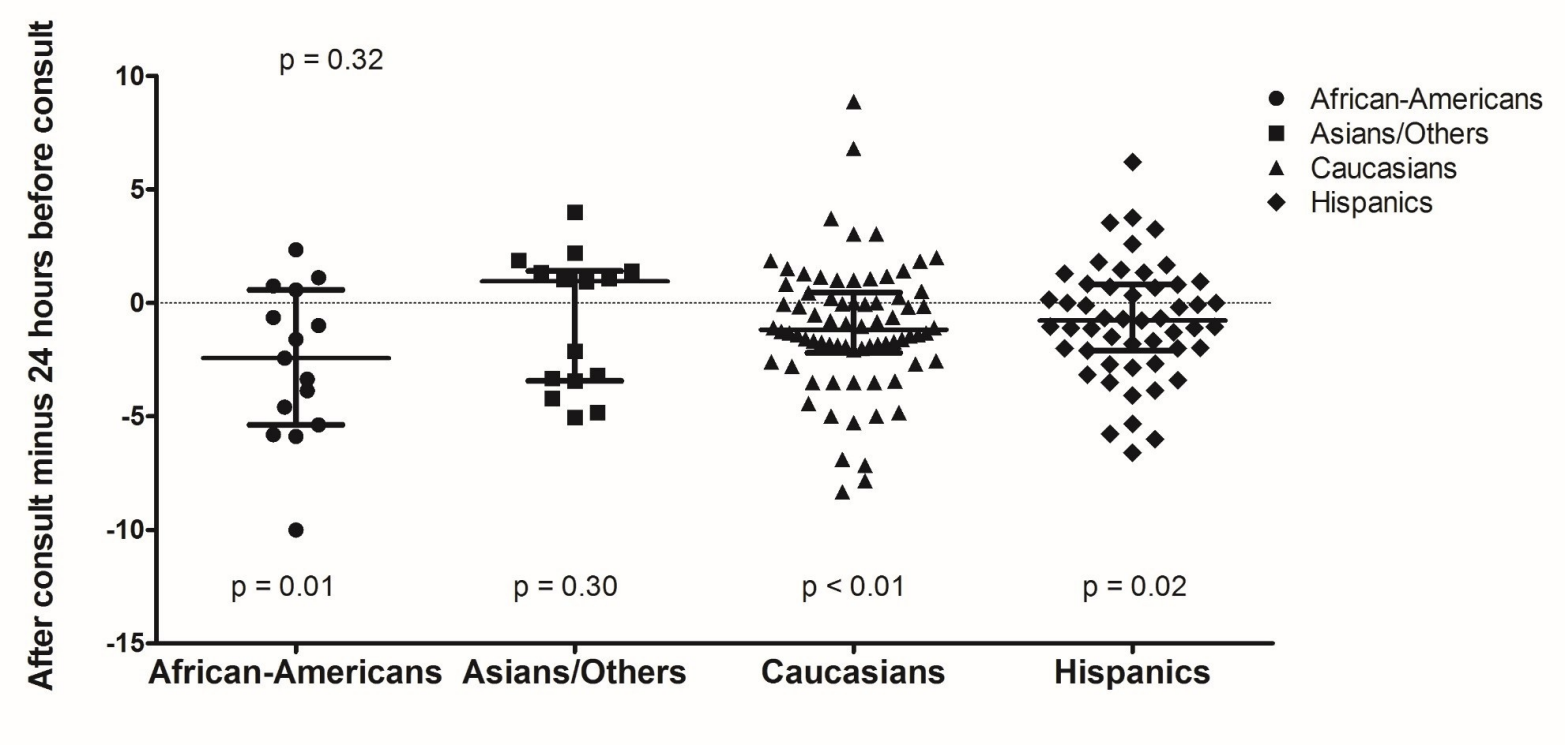
Pain Score Differences After Palliative Consultation Compared to 24 hours Before Consultation by Race/Ethnicity.

**Figure 2:**
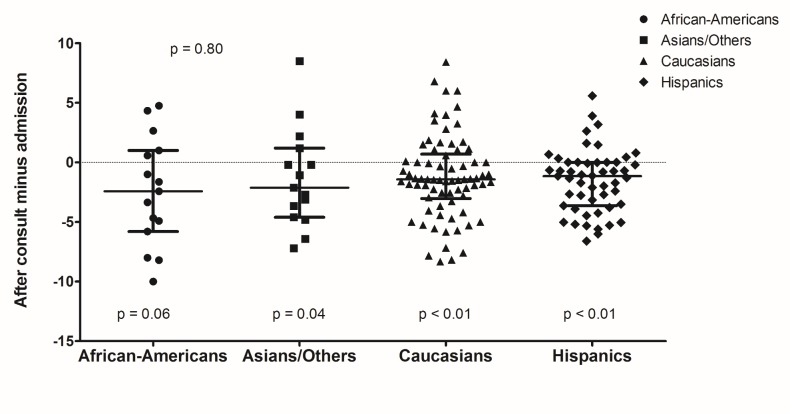
Pain Score Differences After Palliative Consultation Compared to Admission by Race/Ethnicity. .

**Figure 3:**
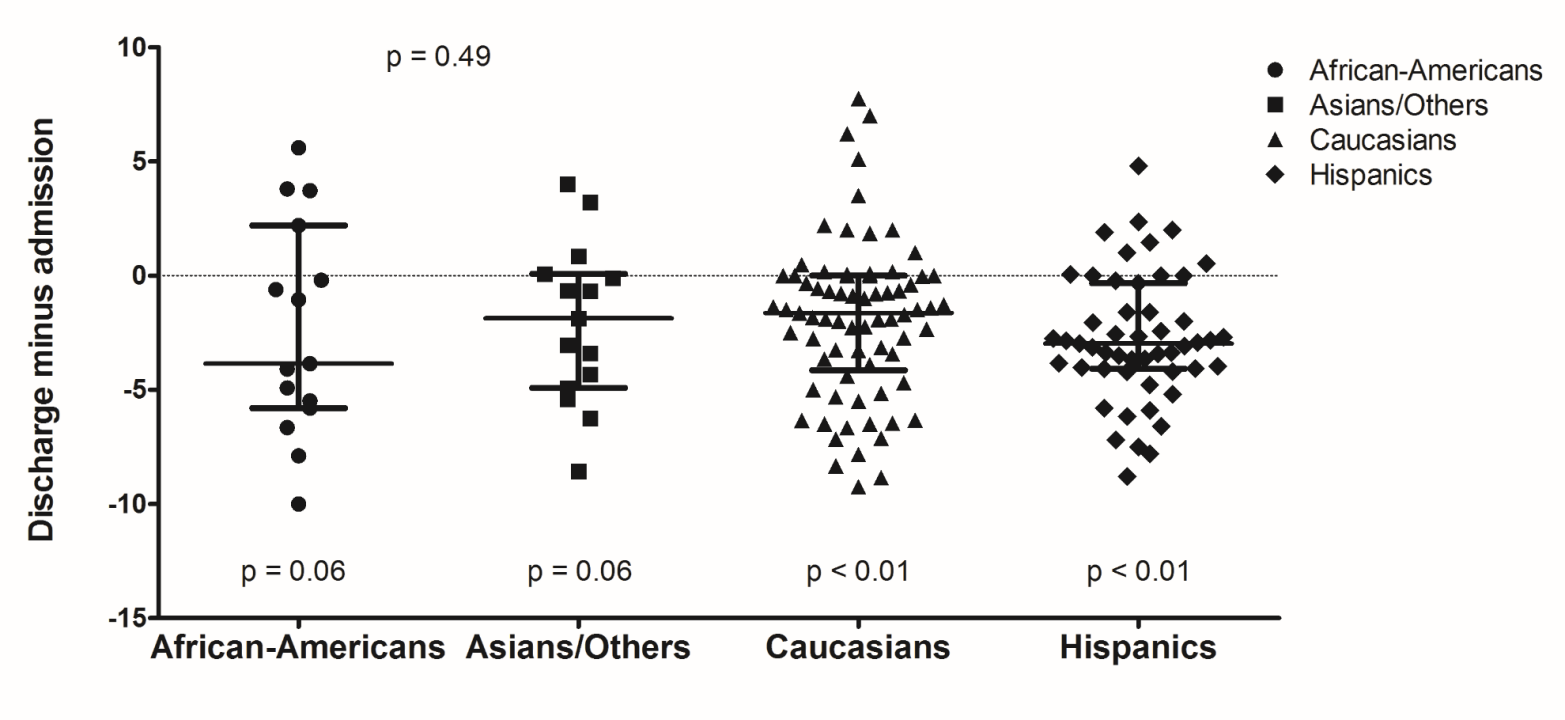
Pain Score Differences at Discharge Compared to Admission by Race/Ethnicity.

African-Americans, Caucasians, and Hispanics had significant reduction in pain scores after consultation compared to 24 hours before (p = 0.01, p < 0.01, and p = 0.02, respectively). Caucasians and Hispanics also had significant differences in pain reduction after palliative consultation compared to initial admission assessment (p < 0.01). Differences between discharge and admission pain scores were significant for Asians, Caucasians, and Hispanics (p = 0.04, p < 0.01, p < 0.01, respectively). Although there were significant pain score differences within certain racial groups, there were overall no significant pain score differences amongst the racial groups [See figures 1-3].

## Discussion

One of the goals of this study was to determine if racial disparities existed in pain outcomes for patients with life-limiting illnesses. A thorough review of the literature by Anderson, et al. determined that ethnic minorities tend to have lower quality pain management in acute, chronic, cancer, and palliative care settings [11]. Overall, we did not find disparity amongst our racial groups.

Asians in our study had non-significant higher initial pain scores compared to the other race/ethnicities. This was in contrast to the findings of Bell, et al. that being of an Asian race increases the likelihood of having lower pain scores when controlling for age, gender, Karnofsky score, and pre-consultation length of stay. Furthermore, the study found better pain outcomes in the non-Caucasian compared to Caucasian groups. It should be noted that the study by Bell, et al. also included surgical patients in addition to patients with cancer and non-cancer diagnoses.

Our study underscores findings of a prior study conducted by Casarett, et al. noting higher overall patient satisfaction scores in the realm of pain and symptom management after palliative care consultation [12]. Although we did not find significant differences between the groups, individual race/ethnicities had significant pain reduction at various time-points.

However, our study had several limitations. First, it was conducted at a single community hospital with potential sample bias reducing generalizability. We only included patients with life-limiting illnesses. Furthermore, our study population was primarily Caucasian and Hispanic; both the African-American and Asian groups together accounted for less than 20% of our population. In addition, male preponderance and age differences could also contribute to the non-homogeneity of the population.There was wide variability in pain scores, but because of the smaller sample size, we lacked the power to find the statistical differences. Cultural influences, different validated pain scales, patient comorbidities, and functional status were not included and could potentially influence the results.

## Conclusions

The present study provides insights into the clinical utility of palliative care services in the inpatient setting. Palliative care consultations have a positive impact on pain reduction. Although there were no significant differences in pain outcomes across the four racial groups, our data showed that pain can be adequately managed regardless of race/ethnicity.
